# Beyond Weight Loss: A Body Composition-Guided Nutrition Framework for Functional Recovery in Heart Failure with Obesity

**DOI:** 10.3390/nu18213683

**Published:** 2026-11-06

**Authors:** Alona D. Angosta, Rebecca Meraz, Sarah Raymond, Joon Jin Song, Henry Han, Lorraine S. Evangelista

**Affiliations:** 1 Louise Herrington School of Nursing, Baylor University, Dallas, TX 75246, USA; rebecca_meraz@baylor.edu; 2 Baylor Scott & White Health, Greater Austin Region, Round Rock, TX 78665, USA; sarah.raymond@bswhealth.org; 3 Department of Statistical Science, Baylor University, Waco, TX 76798, USA; joon_song@baylor.edu; 4 School of Engineering and Computer Science, Baylor University, Waco, TX 76798, USA; henry_han@baylor.edu; 5 Sue & Bill Gross School of Nursing, University of California Irvine, Irvine, CA 92697, USA; l.evangelista@uci.edu

**Keywords:** heart failure, obesity, nutrition therapy, body composition, lean mass preservation, adiposity, sarcopenic obesity, functional recovery, protein intake, quality of life

## Abstract

Heart failure (HF) and obesity commonly occur together and can worsen symptoms, physical function, and quality of life. Although weight loss may benefit selected patients with HF and obesity, body weight alone does not show whether patients are losing fat, muscle, fluid, or a combination of these. This narrative review summarizes selected evidence on nutrition therapy, weight loss, body composition, and functional outcomes in adults with HF, including studies of high-protein diets, nutritional supplements, calorie restriction, Mediterranean-style diets, and obesity pharmacotherapy. Across studies, body composition and functional outcomes have not been measured consistently, making it difficult to determine whether weight loss is associated with meaningful functional improvement. Building on this evidence and findings from the Pro-HEART trial, we propose a body composition-guided nutrition framework that considers changes in fat and lean mass alongside physical function and quality of life. The framework is conceptual and has not yet been validated. Future studies should examine whether nutrition and weight loss interventions can reduce excess fat, preserve muscle, and improve functional and patient-centered outcomes in patients with HF and obesity.

## 1. Introduction

Heart failure (HF) and obesity commonly occur together and can worsen symptoms, physical function, metabolic health, and quality of life. Obesity may contribute to HF through inflammation, insulin resistance, increased hemodynamic burden, and reduced cardiorespiratory fitness [1,2,3]. At the same time, HF can lead to fatigue, reduced physical activity, poor appetite, muscle loss, and nutritional risk [4,5]. These overlapping conditions make nutrition therapy and weight management more complicated.

One reason this issue is complicated is the long-standing “obesity paradox” in HF. Several studies have suggested that patients with higher body mass index (BMI) may have better outcomes than those with lower BMI, even though obesity is a risk factor for cardiovascular disease [3,6]. This has raised questions about whether weight loss should always be encouraged, especially in patients with frailty, malnutrition, or cachexia. However, BMI cannot indicate how much of a patient’s weight is fat, muscle, or fluid [2,7]. A higher BMI may sometimes reflect greater muscle mass or better nutritional status rather than a protective effect of excess fat.

More recently, trials of semaglutide in patients with obesity-related HF with preserved ejection fraction have shown improvements in symptoms, body weight, physical limitations, and walking distance [8,9].

A lower number on the scale may reflect the loss of fat, muscle, fluid, or a combination of these. Losing excess fat may help reduce metabolic stress and make movement easier. Losing muscle may have the opposite effect, leaving patients weaker and less able to function. This is especially important for older adults and patients with diabetes, malnutrition, frailty, or sarcopenic obesity. Sarcopenic obesity involves excess body fat, reduced muscle strength or function, and low muscle mass or changes in muscle composition [10]. Low lean mass alone is not enough to establish sarcopenia. In patients with HF, sarcopenia has been associated with poorer physical function, cardiovascular events, and mortality [4,5,11].

Nutrition is central to this problem, but the evidence is still mixed. Studies have tested high-protein diets, protein supplements, oral nutritional supplements, calorie restriction, Mediterranean-style diets, and diet-plus-exercise approaches in people with HF. Some have focused on patients with obesity, while others have focused on patients with malnutrition, cachexia, or sarcopenia. Our recent systematic review of high-protein dietary interventions in HF found possible benefits for 6-min walk distance, lean body mass, quality of life, and selected clinical outcomes. However, the studies were small and varied in protein dose, intervention length, patient population, and outcome measures [12]. Other nutrition trials show similar limitations, particularly in measuring body composition and determining whether changes in fat or lean mass are associated with improved function [13,14,15].

Pro-HEART is useful in this discussion because it compared a high-protein diet with a standard-protein diet in adults with chronic HF, type 2 diabetes, and overweight or obesity [16,17]. Unlike many nutrition studies, Pro-HEART included measures of body weight, adiposity, lean mass, cardiac structure and function, functional status, and quality of life. These measures provide an opportunity to consider nutrition therapy beyond weight loss alone, although the effects of changes in body composition on functional recovery still need further study.

Our previous systematic review focused specifically on high-protein dietary interventions in HF [12]. The present narrative review looks beyond protein intake to consider nutrition, intentional weight loss, body composition, and physical function together. It includes evidence from lifestyle interventions, nutrition support, and pharmacologic weight loss studies, with an emphasis on patients with obesity or obesity-related risk factors. Based on this evidence, we propose a body composition-guided nutrition framework for HF with obesity. The goal is to help future studies examine not only how much weight patients lose, but also whether they lose excess fat, preserve muscle, and improve physical function and quality of life.

## 2. Literature Identification and Selection

This narrative review builds on our previously published systematic review of high-protein dietary interventions in HF [12]. Details of the databases searched, search dates, and search terms are provided in that publication. We did not conduct a new systematic search for this review, but we included additional studies identified during the development of the body composition-guided nutrition framework.

We selected studies that addressed nutrition, weight loss, body composition, muscle preservation, and physical function in patients with HF. These included clinical trials, systematic reviews, observational studies, and consensus papers. Although our main focus was HF with obesity, we also included studies of patients with malnutrition, cachexia, and sarcopenia because these conditions are vital when considering weight loss and muscle preservation. We focused on studies reporting body weight, fat mass, lean mass, muscle strength, exercise capacity, symptoms, quality of life, or clinical outcomes.

Table 1 summarizes selected original research studies, including the populations studied, interventions, outcomes measured, and remaining gaps. These studies were chosen to illustrate different approaches to nutrition and weight management in HF, as well as the challenges of comparing findings across patient populations. Systematic reviews and consensus statements informed the broader discussion but were not included in Table 1.

## 3. Why Body Weight Alone Is Not Enough in Heart Failure with Obesity

Body weight is easy to measure, but it does not tell the whole story in patients with HF and obesity. A weight change may reflect changes in fat, muscle, fluid, or a combination of these. For example, a patient may lose several pounds after an adjustment to diuretic therapy. Another may lose weight because of poor appetite or muscle loss, while another may lose mostly excess fat. These changes may look similar on the scale, but they have very different clinical meanings.

It is also important to distinguish intentional from unintentional weight loss. Intentional weight loss may benefit selected patients with stable HF and obesity, particularly when the goal is to reduce excess body fat and improve function. Unintentional weight loss is different. It may result from poor appetite, inadequate food intake, cachexia, or worsening HF. For these patients, preventing further weight and muscle loss may be more important than encouraging weight reduction.

Body mass index has similar limitations. Two patients may have the same BMI but very different amounts of fat and muscle, as well as differences in strength and endurance. One may be able to walk and perform daily activities, while another may have fatigue, low muscle mass, and poor physical function. BMI cannot explain these differences or show whether a lower body weight reflects healthier weight loss or declining nutritional status. For this reason, both weight and BMI should be interpreted alongside the patient’s nutritional status, body composition, and physical function.

A body composition approach gives a clearer picture. Instead of asking only whether patients lost weight, clinicians and researchers should ask what type of weight was lost. Did they lose excess fat? Was lean mass preserved? Did walking distance, symptoms, or quality of life improve? These questions matter because fluid status can change quickly in HF. Short-term weight changes may reflect fluid retention or diuresis rather than changes in fat or muscle mass. Daily weight monitoring remains important for detecting fluid retention, but weight alone cannot tell us whether a nutrition or weight loss intervention is helping.

Future HF obesity trials should measure body weight alongside body composition and physical function. These measures may include waist circumference, dual-energy X-ray absorptiometry, bioelectrical impedance analysis, muscle strength, 6-min walk distance, peak oxygen consumption, symptoms, and quality of life. Assessment of muscle strength and function as well as fat and lean mass helps determine sarcopenia.

But these results may not be the whole story. Changes in body composition, physical function, and symptoms could be examined by multivariate analysis. Other statistical techniques, such as factor analysis or composite measures, may also help us detect patterns across outcomes.

## 4. Nutrition, Weight Loss, and Body Composition-Relevant Interventions in Heart Failure: What Has Been Studied So Far

In our recent systematic review, 10 trials involving 1080 patients with HF examined high-protein diets or protein supplementation [12]. The findings suggested possible benefits for functional capacity, lean body mass, quality of life, and selected clinical outcomes. However, the studies varied in protein dose, supplement type, intervention duration, patient population, and measured outcomes. These differences make it difficult to determine which nutrition strategies are most helpful.

Pro-HEART compared a high-protein with a standard-protein calorie-restricted diet in adults with chronic HF, type 2 diabetes, and overweight or obesity [16,17]. After 3 months, both diets produced similar reductions in body weight (3.6 versus 2.9 kg). However, the high-protein group had greater reductions in glycated hemoglobin (0.7% versus 0.1%, *p* = 0.002), total cholesterol (16.8 versus 0.9 mg/dL, *p* = 0.031), and triglycerides (25.7 versus 5.7 mg/dL, *p* = 0.032). The trial included measures of adiposity and cardiometabolic outcomes, but these findings do not establish whether changes in body composition led to better physical function.

Kitzman et al. [18] studied 100 older adults with obesity and stable HF with preserved ejection fraction. Over 20 weeks, calorie restriction and aerobic exercise improved peak oxygen uptake by 1.3 and 1.2 mL/kg/min, respectively (both *p* < 0.001). Combining the interventions produced an additive improvement of 2.5 mL/kg/min. Changes in peak oxygen uptake were also correlated with changes in percentage lean body mass (r = 0.32, *p* = 0.003). These findings suggest that body composition may be relevant to functional improvement, although the relationships do not establish causation.

Studies involving malnourished or nutritional at-risk patients provide a different perspective. In the PICNIC trial, which included 120 hospitalized patients with HF and malnutrition, 12-month mortality was 20.3% in the nutrition intervention group versus 47.5% in the control group (*p* = 0.003) [13]. In the EFFORT HF subgroup analysis involving 645 hospitalized patients, 30-day mortality was 8.4% with individualized nutritional support compared with 14.8% with standard hospital food (adjusted odds ratio, 0.44; 95% CI, 0.26–0.75) [14]. These studies involved patients at nutritional risk rather than patients undergoing intentional weight loss, and their findings should not be interpreted as evidence supporting weight reduction in these populations.

Other studies have examined body composition more directly. Dos Santos et al. [19] conducted a randomized, placebo-controlled trial in 33 patients with chronic HF, of whom 25 completed the 12-week intervention. Patients receiving 30 g/day of whey protein isolate had an increase in skeletal muscle mass of 0.6 ± 0.7 kg, along with reductions in waist circumference (*p* = 0.001) and body fat percentage (*p* = 0.026). Skeletal muscle index also increased (*p* = 0.004), but handgrip strength did not improve significantly. These findings suggest that protein supplementation may improve body composition without necessarily improving muscle strength. Herrera-Martínez et al. [15] studied 38 patients with HF receiving a Mediterranean diet and vitamin D, with the intervention group receiving additional hypercaloric, hyperproteic oral supplementation. Lean mass increased by 1.2 kg in the intervention group (*p* = 0.03). Although functional measures improved during follow-up, these findings came from a small trial and do not establish that changes in lean mass caused the functional improvements.

Pharmacologic weight loss should be considered separately from nutrition interventions. In STEP-HFpEF, patients receiving semaglutide had a mean weight reduction of 13.3%, compared with 2.6% with placebo, and a greater improvement in 6-min walk distance (between-group difference 20.3 m, *p* < 0.001) [8]. In STEP-HFpEF DM, weight decreased by 9.8% with semaglutide compared with 3.4% with placebo, with a 14.3 m between-group improvement in walking distance (*p* = 0.008) [9]. Although these trials demonstrated benefits for weight loss and physical function, they did not establish how much of the weight loss was fat versus muscle. Future studies should examine body composition during medication-assisted weight loss, including whether adequate protein intake and physical activity help preserve muscle.

Overall, these studies show that nutrition and weight loss interventions may affect more than body weight. However, differences in patient populations, interventions, and outcomes make the findings difficult to compare. Few studies have examined whether changes in fat and lean mass are related to improvements in physical function. This remains an important gap in HF nutrition research.

## 5. What This Evidence Shows and What It Still Misses

The studies summarized in Table 1 suggest that nutrition and weight loss interventions in HF may influence more than just body weight. However, the studies had varied patient populations, interventions, and follow-up periods [12,18]. They also measured multiple outcomes, making it difficult to compare findings or determine which approaches work best for certain patients.

The strength of the evidence also varies. Randomized clinical trials provide evidence about specific interventions, although some included small or selected patient populations. Systematic reviews synthesize findings from multiple studies, but their conclusions depend on the quality of the included studies. Subgroup analyses should be interpreted with caution, while consensus statements provide guidance rather than direct evidence of treatment effects. These differences are important when interpreting the findings.

Another problem is that many studies measured outcomes separately. Some measured body weight but not body composition, while others measured fat or lean mass without examining changes in physical function [8,9,16,19]. As a result, we still do not know enough about whether changes in body composition help explain improvements in symptoms, walking distance, or quality of life. This matters because patients with HF want to do more than lose weight. They want to feel better, remain independent, and manage their daily activities.

Differences between studies also make it difficult to combine their findings. Future reviews may consider statistical approaches to account for differences in HF populations, interventions, and outcomes. Random-effects meta-analysis may be useful when studies are adequately comparable; meta-regression and multivariate methods may be useful for investigating differences in study characteristics and relationships among outcomes. These methods may provide a better understanding of how nutrition and weight loss interventions affect body composition and physical function.

## 6. Body Composition as a Nutrition-Relevant Clinical Target

Body composition provides information that body weight cannot. A patient may lose fat, muscle, fluid, or some combination of these. Losing excess fat may help reduce metabolic stress and make movement easier. Losing muscle is different. It can leave patients weaker and less able to walk, exercise, or recover from illness [10,20].

There are several ways to assess body composition. Dual-energy X-ray absorptiometry can estimate fat mass, lean mass, and percent body fat [11,21]. Bioelectrical impedance analysis is easier to use in many clinical settings, but results can be affected by fluid status [22]. Waist circumference is a simple measure of central adiposity, but it does not indicate a patient’s muscle mass [23]. No single measure is perfect, and the choice depends on the patient’s condition and available resources.

Daily weight monitoring is still important in HF because it helps detect fluid retention. However, when evaluating nutrition or weight loss interventions, we also need to look at body composition, muscle strength, and physical function. These measures can help us understand whether patients are losing excess fat, keeping enough muscle, and functioning better.

## 7. Preserving Lean Mass While Reducing Excess Adiposity

The aim should not be weight loss at any cost for patients with HF and obesity. If weight loss can improve symptoms or function, it may be helpful if it results in loss of excess fat. But this may not be helpful if the patient also loses significant muscle mass [10,23]. This is why preserving muscle needs to be part of the conversation from the beginning.

This is particularly important in HF, where many patients already have fatigue, low levels of activity, poor exercise tolerance, and risk for frailty or sarcopenia [4,10,23]. Some may also have poor appetite, dietary restrictions, diabetes, kidney disease, or lack of access to healthy foods. These issues can make advice on losing weight harder to follow. But telling someone ‘eat less and lose weight’ is a general message that may not be sufficient or may even be risky for some patients.

Protein intake might help preserve muscle during weight loss, but HF patients do not all have the same protein needs. Older adults and patients with frailty, sarcopenia, or poor nutrition may require attention to protein intake. Different amounts are needed depending on kidney function and whether or not the patient has dialysis. In patients with advanced HF, poor appetite, or cachexia, the prevention of further muscle and weight loss may be more important than trying to lose weight. Protein recommendations should therefore consider the patient’s age, kidney function, nutritional status, frailty, HF severity, and treatment goals [12,24].

Physical activity is also important. Protein alone may not be enough to protect muscle. Current HF guidelines recommend exercise training or regular physical activity for patients who can participate safely to improve functional status, exercise performance, and quality of life [25]. Resistance training, walking, and cardiac rehabilitation may also help patients maintain strength and function during weight loss.

Nutrition support also needs to be practical. Many patients with HF are asked to follow several dietary recommendations, including sodium restriction, fluid management, diabetes-related dietary changes, and weight management. Adding protein goals, calorie targets, or supplements may feel overwhelming. A plan that patients cannot follow in daily life is unlikely to improve body composition or function.

Anti-obesity medications add another layer to this issue. These therapies can produce substantial weight loss and may improve symptoms and function in selected patients with obesity-related HF [8,9]. However, we still need to know whether patients are losing mostly fat, preserving enough muscle, and functioning better. This is particularly important in older adults and in patients at risk of frailty or sarcopenic obesity.

You may need sufficient protein intake, safe physical activity, attention to other health conditions, and support for behavior change to preserve muscle while losing excess fat. In HF, the goal should be weight loss that helps the patient, not weight loss that leaves the patient weaker.

## 8. A Body Composition-Guided Framework for HF Nutrition and Functional Recovery

**Figure 1:**
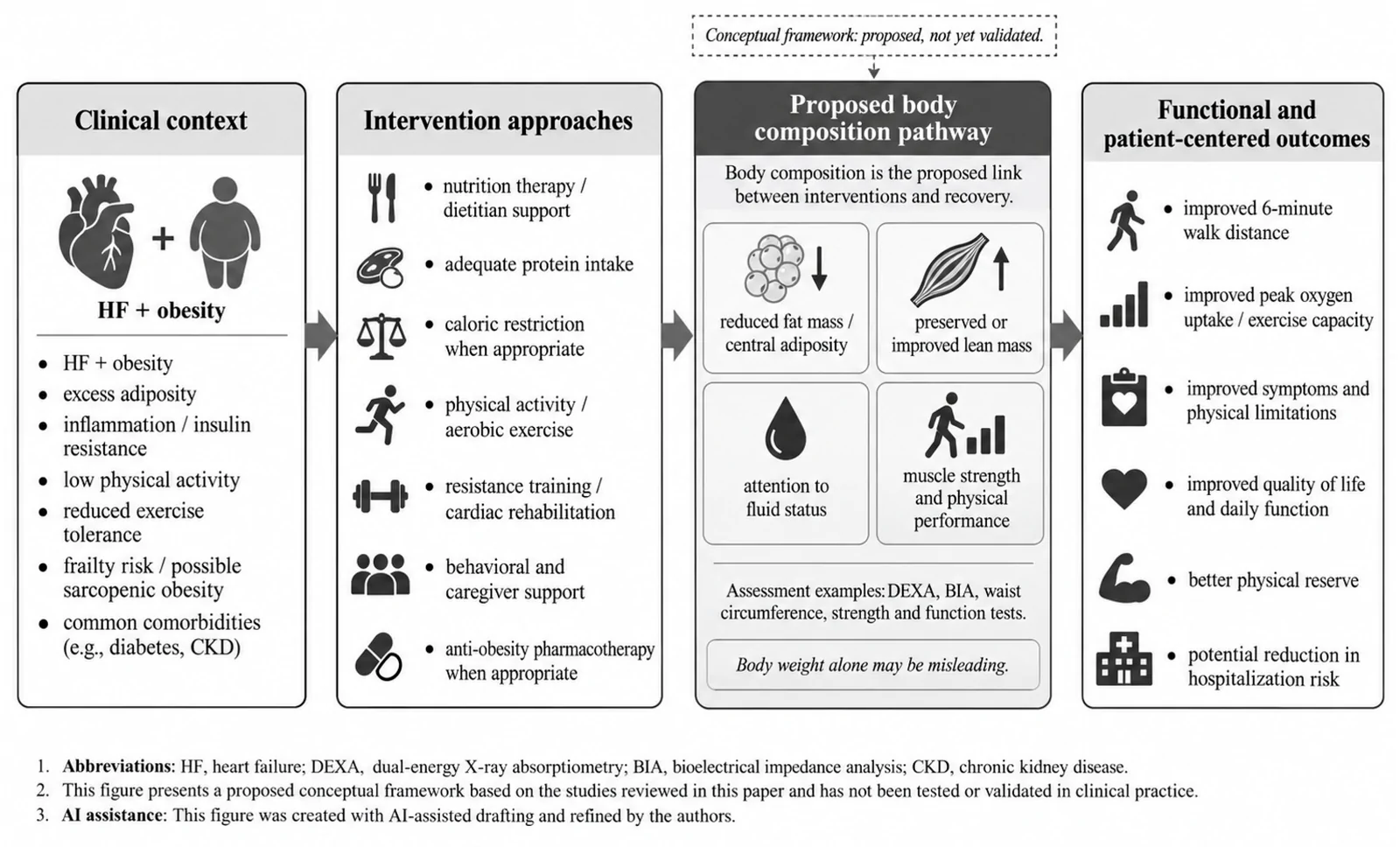
Body composition-guided framework for nutrition and functional recovery in heart failure with obesity.

Figure 1 presents a proposed body composition-guided framework for HF nutrition and functional recovery. The framework is based on the evidence reviewed and has not yet been tested or validated [12,17,18]. It begins with the challenges associated with HF and obesity, including inflammation, insulin resistance, reduced physical activity, poor exercise tolerance, frailty, and sarcopenic obesity. These problems may affect how patients respond to nutrition and weight loss interventions.

The intervention approaches in the proposed framework include nutritional therapy, adequate protein intake, physical activity, resistance training, behavioral support, and anti-obesity drugs, when indicated. Treatment decisions should be based on the patient’s condition, other health problems, and preferences and goals. For example, a patient with obesity-related HFpEF and diabetes may need a different approach than a patient with advanced HF, poor appetite, or frailty.

The center of the framework is body composition. Rather than focusing only on how much weight patients lose, we also need to know whether they lose excess fat, preserve muscle, or experience changes that may worsen frailty and function. What was lost can make the impact of losing the same amount of weight different.

The final part focuses on outcomes that matter to patients, including walking distance, exercise capacity, muscle strength, HF symptoms, quality of life, hospitalization, and ability to perform daily activities [8,9,18]. Changes in body composition may help explain why some patients improve after treatment while others do not [25]. However, this relationship still needs to be tested.

The framework also recognizes that treatment goals may differ among patients. Some may benefit from reducing excess fat, while others may need to prevent further muscle loss. For others, the priority may be walking farther, feeling less tired, or remaining independent. The goal is to help researchers examine not just how much weight patients lose, but what they lose and whether they function better.

## 9. Implications for Future HF Nutrition and Weight Loss Trials

Future HF nutrition and weight loss trials need to measure more than weight change. A patient may lose weight and still become weaker. Another may lose less weight but preserve muscle, walk farther, and feel better. Future trials should be designed to capture these differences.

One priority is to measure body composition more consistently. Trials should include fat mass, lean mass, and central adiposity when feasible. Dual-energy X-ray absorptiometry can estimate fat and lean mass, while bioelectrical impedance analysis may be more practical in clinical settings but can be affected by fluid shifts. Waist circumference is another useful measure of central adiposity. These measures should be used alongside body weight to help explain what patients are losing.

Future trials should also measure physical function, symptoms, and quality of life. The 6-min walk test, peak oxygen consumption, gait speed, grip strength, chair stands, frailty measures, and patient-reported physical limitations are useful. Patients with HF may experience fatigue, shortness of breath, and difficulty with daily activities. Measuring these outcomes can help determine whether an intervention improves how patients feel and function, not just their weight.

Emerging analytic and digital tools may also be helpful. Artificial intelligence-assisted imaging and wearable monitoring may eventually allow researchers to examine body composition, fluid status, and physical function together. Multivariate and other statistical approaches may help identify patterns across these outcomes. However, these methods still need to be tested in HF nutrition and weight loss studies and should not replace clinical assessment.

Nutrition interventions also need to be described clearly. Studies should report protein and calorie targets, dietary patterns, supplements, physical activity recommendations, behavioral support, and adherence. Researchers should also consider differences among HF populations. Patients with obesity-related HFpEF may have different needs from those with advanced HF, malnutrition, cachexia, or sarcopenic obesity. These differences should be considered when deciding whether an intervention is safe and appropriate.

Finally, future studies should test combined approaches that include nutrition, physical activity, resistance training, cardiac rehabilitation, behavioral support, and, when appropriate, anti-obesity medications. They should also test the proposed framework to determine whether changes in body composition help explain improvements in physical function and quality of life. The goal is to identify approaches that help patients lose excess fat when appropriate, preserve muscle, and function better.

## 10. Conclusions

This review brings together evidence on nutrition, weight loss, body composition, and functional outcomes in patients with HF and obesity. Based on this evidence, we propose a body composition-guided framework that considers whether patients lose excess fat, preserve muscle, and improve physical function and quality of life. Although the framework has not yet been validated, it may help guide future studies toward identifying nutrition and weight loss approaches that lead to better outcomes for patients with HF and obesity.

## Figures and Tables

**Table 1:** **Selected Original Research Studies on Nutrition, Weight Management, Body Composition, and Functional Outcomes in Heart Failure.** *Research studies only; organized by primary clinical focus. Numbers are drawn from the cited original publications*.

Study (Reference)/Design	HF Population/Sample	Intervention or Exposure	Selected Numerical Findings	Interpretation for Framework
**INTENTIONAL WEIGHT LOSS/LIFESTYLE**				
Pro-HEART: Evangelista et al. (2021) [16]; randomized trial	Overweight/obesity, HF and diabetes; n = 76	Calorie-restricted high-protein vs. standard-protein diets; 3 months	Weight reduction: 3.6 vs. 2.9 kg (between groups not significant); HbA1c reduction: 0.7 vs. 0.1 percentage points (*p* = 0.002).	Weight loss was comparable; cardiometabolic changes differed. No demonstrated body-composition mediation of function.
Kitzman et al. (2016) [18]; randomized 2 × 2 factorial trial	Older adults with obesity and stable HFpEF; n = 100	Calorie restriction, aerobic exercise, both, or control; 20 weeks	Peak VO_2_ improvement: ~1.3 mL/kg/min with diet and ~1.2 with exercise; combination ~2.5 mL/kg/min.	Functional improvements support assessing exercise and dietary change together; mechanism not established.
**MALNUTRITION/NUTRITION SUPPORT**				
PICNIC: Bonilla-Palomas et al. (2016) [13]; randomized trial	Hospitalized HF with malnutrition; n = 120	Individualized nutritional intervention plus usual care vs. usual care	12-month death: 20.3% vs. 47.5% (p = 0.003); HF readmission: 10.2% vs. 36.1% (*p* = 0.001).	Supports nutritional care for malnourished HF patients; no evidence for intentional weight loss.
EFFORT HF: Hersberger et al. (2021) [14]; HF subgroup of randomized trial	Hospitalized chronic HF at nutritional risk; n = 645	Individualized nutritional support vs. standard hospital meals	30-day mortality: 8.4% vs. 14.8%; adjusted OR 0.44 (95% CI 0.26–0.75).	Distinct inpatient nutritional-risk population; subgroup evidence, not weight-loss evidence.
**MUSCLE PRESERVATION/BODY COMPOSITION**				
dos Santos et al. (2023) [19]; randomized, placebo-controlled trial	Chronic HF; trial enrolled 25 patients for final analysis	Whey protein isolate 30 g/day vs. placebo; 12 weeks	Skeletal muscle mass/index increased, and body fat percentage and waist circumference decreased; no significant muscle strength benefit.	Supports measuring muscle quantity and strength separately; small study.
Herrera-Martínez et al. (2024) [15]; randomized trial	HF with sarcopenia; n = 38	Mediterranean diet and vitamin D, with added hypercaloric/high-protein supplements in intervention group	Intervention-group lean-mass gain reported as ~1.2 kg (*p* = 0.03); interpretation requires attention to between-group results.	Suggests lean-mass responsiveness; small sample and multiple co-interventions.
Saito et al. (2022) [20]; observational FRAGILE-HF analysis	Older patients hospitalized with HF	Assessment of sarcopenic obesity and subsequent outcomes	Reported associations with impaired physical function and mortality; no randomized treatment contrast.	Shows prognostic relevance; cannot infer benefits of an intervention.
Saito et al. (2024) [11]; observational prognostic study	Older HF patients	DXA-based sarcopenia assessment and prognostic modeling	Assessed relationships of DXA-derived muscle measures with prognosis; not an intervention trial.	Supports measuring body composition as risk information, not proof of intervention effect.
Salmons et al. (2025) [21]; observational comparison study	Patients with obesity-related HFpEF	Cross-sectional body composition comparison	Characterized differences in body composition; no randomized weight-loss or functional-recovery intervention.	Informs HFpEF phenotype differences; not causal evidence.
**PHARMACOLOGIC OBESITY TREATMENT**				
STEP-HFpEF: Kosiborod et al. (2023) [8]; randomized trial	Obesity-related HFpEF without diabetes; n = 529	Semaglutide 2.4 mg weekly vs. placebo; 52 weeks	Weight change: −13.3% vs. −2.6%; between-group 6-minute-walk improvement: +20.3 m.	Improved weight and functional endpoints; body-composition mediation not established.
STEP-HFpEF DM: Kosiborod et al. (2024) [9]; randomized trial	Obesity-related HFpEF with type 2 diabetes; n = 616	Semaglutide 2.4 mg weekly vs. placebo; 52 weeks	Weight change: −9.8% vs. −3.4%; between-group 6-minute-walk improvement: +14.3 m.	Supports symptom/function benefit in the diabetes subgroup; muscle-preservation effects not established.

Notes: HF = heart failure; HFpEF = heart failure with preserved ejection fraction; DXA = dual-energy X-ray absorptiometry; HbA1c = glycated hemoglobin; OR = odds ratio; CI = confidence interval; VO_2_ = oxygen uptake. Numerical results are selected outcomes, not a quantitative synthesis. The Pro-HEART design paper [17] is not listed separately to avoid counting one trial twice. Consensus statements, systematic and narrative reviews, and clinical guidelines were excluded from this table.

## Data Availability

No new data were created or analyzed in this study. Data sharing is not applicable to this article.

## Abbreviations

The following abbreviations are used in this manuscript:

Abbreviation	Term
BMI	Body mass index
HF	Heart failure
HFpEF	Heart failure with preserved ejection fraction
QOL	Quality of life
